# Sarcoidosis in a 65-year-old woman presenting with a lung mass and pericardial effusion: a case report

**DOI:** 10.1186/1752-1947-6-259

**Published:** 2012-08-31

**Authors:** George A Margaritopoulos, Athanasia Proklou, Eleni Lagoudaki, Argiro Voloudaki, Nikos M Siafakas, Katerina M Antoniou

**Affiliations:** 1 Department of Thoracic Medicine, Interstitial Lung Disease Unit, University Hospital of Heraklion, Panepistimiou Avenue, Voutes, 71110, Heraklion, Crete, Greece; 2 Department of Pathology, Medical School of the University of Crete, Panepistimiou Avenue, Voutes, 71110, Heraklion, Crete, Greece; 3 Department of Radiology, University Hospital of Heraklion, Panepistimiou Avenue, Voutes, 71110, Heraklion, Crete, Greece

**Keywords:** Sarcoidosis, Lung cancer, Pleural effusion, Pericardial effusion.

## Abstract

**Introduction:**

Sarcoidosis is a multi-systemic disorder of unknown origin and most commonly affects the lungs. Diagnosis relies on the presence of non-caseating granulomas on histologic specimens. In high-resolution computed tomography, the most characteristic findings are peribronchovascular thickening, perilymphatic nodular distribution, and bilateral hilar adenopathy. Confluent nodular opacities or large masses are rare manifestations of the disease. It is well recognized that sarcoidosis can mimic infectious, malignant, and granulomatous conditions. Here, we report a case with a high initial index of suspicion for lung malignancy in terms of clinical, lung imaging, and endoscopic findings.

**Case presentation:**

A 65-year-old Caucasian woman, lifelong non-smoker with an unremarkable medical history, presented with a 10-month history of progressive breathlessness, dry cough, fatigue, arthralgias, and mild weight loss. The only significant clinical finding was bilateral enlargement of auxiliary lymph nodes. High-resolution computed tomography revealed a soft tissue density mass at the right hilum which was surrounding and narrowing airways and vascular components, nodules with vascular distribution, enlarged mediastinal lymph nodes, and pericardial effusion. Our patient underwent a bronchoscopy, which revealed the presence of submucosal infiltration and narrowing of the right upper bronchus. Endobronchial biopsies showed non-caseating granulomas. As local sarcoid reactions with non-caseating granulomas can be observed near tumors, our patient underwent video-assisted thoracoscopy and surgical removal of an auxiliary lymph node, both of which confirmed the presence of non-caseating granulomas and the diagnosis of sarcoidosis. She was treated with steroids with improvement of clinical and imaging findings. However, while on a maintenance dose, she presented with a pleural effusion, which, after the diagnostic work-up, proved to be sarcoidosis-related. Treatment with initially high doses of steroids plus a steroid-sparing agent led to resolution of the effusion.

**Conclusions:**

We report a case with a high initial index of suspicion for lung malignancy. Clinicians should always be aware that sarcoidosis enters the differential diagnosis of patients presenting with a lung mass that encases and narrows bronchial and vascular structures with associated pericardial effusion. Rarely, pleural effusion can be the presenting symptom of disease relapse despite maintenance treatment.

## Introduction

Sarcoidosis is a multi-systemic disease in which inflammatory cells gather and form nodules known as non-caseating epithelioid granulomas. The most commonly affected organs are the lungs, eyes, and skin, but all the organs can be potentially affected. The diagnosis is established when clinico-radiographic findings are supported by histologic evidence of non-caseating granulomatous inflammation and other causes of granulomas and local reactions have been reasonably excluded [1]. In recent years, high-resolution computed tomography (HRCT) has assumed a key role in the diagnosis of the disease. The most characteristic findings are peribronchovascular thickening, perilymphatic nodular distribution, and bilateral hilar adenopathy. Confluent nodular opacities, present on HRCT as areas of lung consolidation with air bronchograms or as large masses, are rare manifestations of the disease. The main differential includes lymphangitic carcinomatosis [2]. Sarcoidosis has an upper-lobe predominance, whereas lymphangitic carcinomatosis shows a more extensive and linear thickening of the interlobular septa and frequently is unilateral whereas sarcoidosis typically is bilateral in nature. The differential diagnosis with other nodular diseases is based on the study of the distribution pattern and the delineation of the nodules [3,4]. Though sharply defined, hematogenous metastases usually show a random distribution. Miliary infection (tuberculosis and fungus) shows a random distribution, and nodules can be well or ill defined. Hypersensitivity pneumonitis, bronchiolitis, and hemorrhage can also have a nodular appearance but these nodules are usually ill defined and located in the center of the secondary pulmonary lobule. Nodules seen in organizing pneumonia and Langerhans cell histiocytosis are usually well defined and also show a centrilobular distribution. Large unilateral masses are rare manifestations. Occasionally, sarcoidosis can simulate idiopathic pulmonary fibrosis [5], although honeycombing is seldom striking. We present a case of sarcoidosis in which the most likely initial diagnosis based on clinical, radiological, and macroscopic findings on bronchoscopy was lung malignancy.

## Case presentation

A 65-year-old Caucasian woman, lifelong non-smoker with an unremarkable medical history, presented with a 10-month history of progressive breathlessness, dry cough, fatigue, arthralgias, and mild weight loss. A physical examination revealed only the presence of palpable auxiliary lymph nodes bilaterally. Laboratory tests showed a white blood cell count of 4.7K/μl, a low lymphocyte count of 0.8K/μl, and an increased erythrocyte sedimentation rate of 42mm/h. Autoantibody profile, C-reactive protein, and angiotensin-converting enzyme were negative. Lung function tests showed an obstructive pattern with a forced expiratory volume in the first second (FEV1) of 66.8%, a forced vital capacity (FVC) of 83.4%, and a mild reduction of carbon monoxide diffusing capacity (DLco), which was 69.7%. Mantoux test was negative.

**Figure 1:**
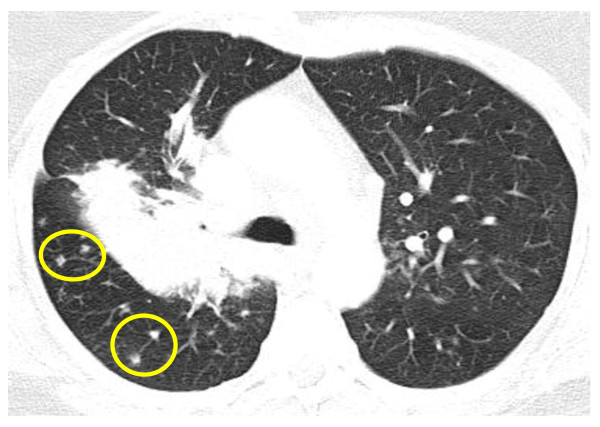
**High-resolution computed tomography image of the chest at presentation (upper-lung zones).** A mass-like lesion with nodules in the right lung (yellow circles) is shown.

**Figure 2:**
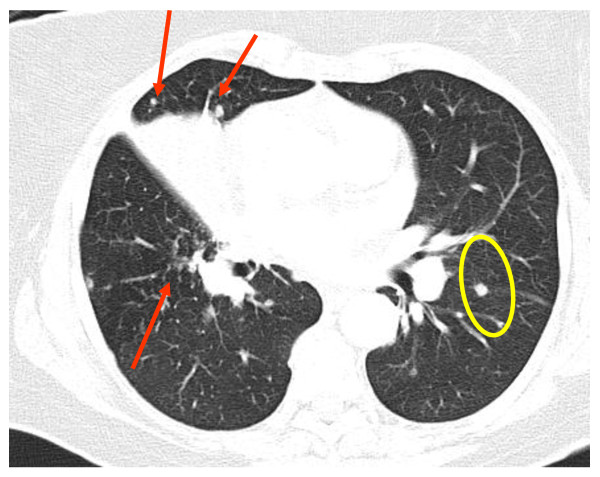
**High-resolution computed tomography image of the chest at presentation (middle-lung zones).** A mass-like lesion with nodules in the right (red arrows) and left (yellow circles) lung is shown.

As revealed by HRCT, a soft tissue density mass at the right hilum was extending into the right upper lobe, surrounding and narrowing the lower part of the trachea, the right main bronchus, and its lobar branches, and encasing the right pulmonary artery. In addition, there were nodules of up to 1cm in dimension with vascular distribution in upper- and mid-lung zones bilaterally, enlarged paratracheal, aortopulmonary, mediastinal, and auxiliary lymph nodes, and pericardial effusion of medium amount (Figures 1 and 2). These findings were described as being consistent with bronchogenic carcinoma.

We proceeded with bronchoscopy, which revealed submucosal infiltration and narrowing of the right upper bronchus. The results of cultures, stains, and cytology were negative. Surprisingly, endobronchial biopsies showed non-caseating granulomas.

**Figure 3:**
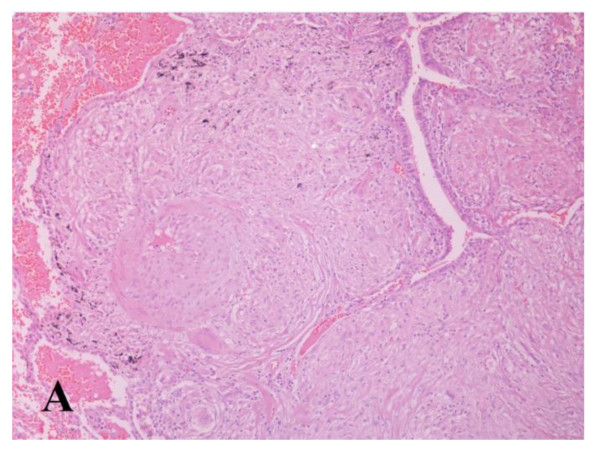
**Histologic picture of video-assisted thoracoscopy lung biopsy sample.** The submucosa of the bronchus shows confluent non-caseating granulomas, which narrow the bronchial lumen. Stain: hematoxylin and eosin; magnification: ×200.

Faced with a non-specific finding such as granulomatous inflammation in a patient with lobar atelectasis, pericardial effusion, and non-specific constitutional and respiratory symptoms, we proceeded to surgical removal of an auxiliary lymph node and video-assisted thoracoscopy (VATS) to exclude with certainty the presence of malignancy. Histology of the lung specimen revealed multiple, confluent, well-formed, non-caseating granulomas (Figure 3). The excised lymph node also showed non-caseating granulomas. The results of stains for acid-fast bacteria, fungi, and microorganisms in both specimens were negative.

**Figure 4:**
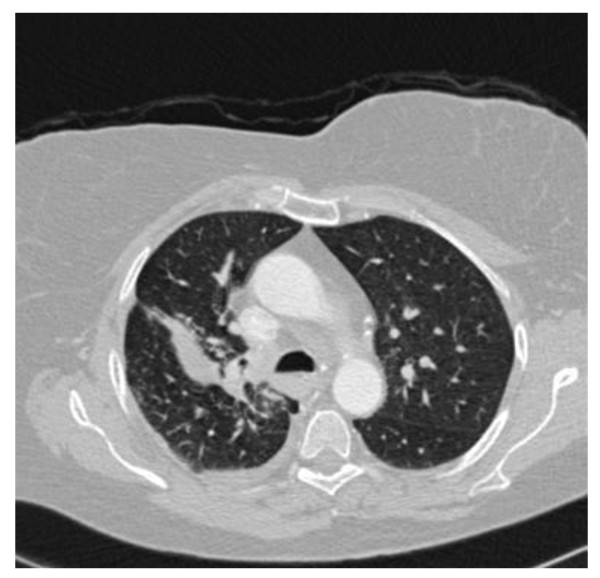
**High-resolution computed tomography scan of the chest (upper-lung zones).** Radiological improvement was achieved after treatment with steroids was commenced.

The histologic features mentioned above were consistent with sarcoidosis, and treatment with high doses of oral steroids was commenced. Clinical and radiological improvement was achieved (Figure 4). Interestingly six months later, while on a maintenance treatment with 10mg of prednisolone, she presented with a right-side pleural effusion. Thoracentesis showed an exudate with 85% lymphocytes. In light of the results of diagnostic tests, other causes of pleural effusion such as infection or malignancy were excluded, and the working diagnosis was sarcoidosis-related pleural effusion. Our patient was treated initially with high doses of oral prednisolone with gradual tapering plus a steroid-sparing agent, methotrexate, and complete regression of the pleural effusion was observed in follow-up visits.

## Discussion

Dominant mass-like nodular sarcoidosis or isolated nodules mimicking malignancy or infection are rare manifestations of sarcoidosis [6]. Lobar atelectasis, due to endobronchial nodules or enlarged adjacent lymph nodes, is surprisingly uncommon, mainly given the frequency of lymph node enlargement in sarcoidosis, and only a few cases have been reported [7]. Non-caseating granulomas observed in biopsy samples provide confidence in the diagnosis of sarcoidosis but only in the right clinical setting [7]. Also, local sarcoid reactions with non-caseating granulomas in or near tumors and, less frequently, in regional draining lymph nodes are commonly reported in lymphomas and breast, primary lung, renal cell, ovarian, and stomach cancers [8]. It is recommended that when an atypical presentation is encountered, biopsies from two non-contiguous sites be conducted to provide a definitive diagnosis [6]. In this case, both VATS and lymph node biopsy excluded malignancy and confirmed sarcoidosis. Initially, the suspicion of sarcoidosis was low and thus bronchoalveolar lavage and transbronchial biopsies were not performed.

The treatment decision was based on impaired quality of life (fatigue, weight loss, and arthralgias) and respiratory symptoms (breathlessness). The treatment of pericardial effusion, when asymptomatic, is controversial. One study demonstrated that sarcoidosis with asymptomatic cardiac involvement has an excellent long-term prognosis without therapy. However, only three asymptomatic patients out of 82 patients were screened, making this conclusion unreliable [9].

Pleural effusion, the presenting symptom of disease relapse when our patient attempted to reduce the dose of steroids, is an uncommon manifestation of sarcoidosis (the estimated incidence is 0.7% to 10%) and is more common in patients with active parenchymal disease. Suggested mechanisms are presumably similar to those of other infiltrative diseases. Involvement of the pleura may lead to increased capillary permeability with minimal pleural space inflammation [10]. In a recent study of patients with sarcoidosis, pleural effusion was diagnosed in five out of 181 cases by using chest ultrasound, whereas a biopsy-proven diagnosis was made in only two cases [11]. The effusions are usually small to moderate in volume, tend to affect the right side more often than the left, and are lymphocytic exudates. T-lymphocyte CD4/CD8 ratio is usually elevated. It is essential to exclude infections or malignancies as possible causes, and a firm diagnosis can be obtained via pleural biopsy. Usually, the effusions resolve spontaneously or after treatment with steroids.

## Conclusions

It is well recognized that sarcoidosis can mimic infectious, malignant, and granulomatous conditions and may predispose patients to the development of lung cancer [12]. Here, we report a case with a high initial index of suspicion for lung malignancy in terms of clinical, lung imaging, and endoscopic findings. Clinicians should always be aware that sarcoidosis enters the differential diagnosis of patients presenting with a lung mass that infiltrates, encases, and narrows bronchial and vascular structures.

## Consent

Written informed consent was obtained from the patient for publication of this case report and accompanying images. A copy of the written consent is available for review by the Editor-in-Chief of this journal.

## Abbreviations

HRCT, High-resolution computed tomography; VATS, Video-assisted thoracoscopy.

## Competing interests

The authors declare that they have no competing interests.

## Authors’ contributions

GAM performed the physical examination of the patient, analyzed clinical and laboratory findings, and was a major contributor in writing the manuscript. AP performed the bronchoscopy. EL performed the pathologic review of the lung and auxiliary lymph node specimens. AV interpreted the HRCT images. NMS and KMA analyzed the manuscript for important intellectual content. All authors read and approved the final manuscript.
